# Sensitivity of cholecystokinin receptors to membrane cholesterol content

**DOI:** 10.3389/fendo.2012.00123

**Published:** 2012-10-18

**Authors:** Aditya J. Desai, Laurence J. Miller

**Affiliations:** Department of Molecular Pharmacology and Experimental Therapeutics, Mayo ClinicScottsdale, AZ, USA

**Keywords:** cholecystokinin, G protein-coupled receptors, cholesterol

## Abstract

Cholesterol represents a structurally and functionally important component of the eukaryotic cell membrane, where it increases lipid order, affects permeability, and influences the lateral mobility and conformation of membrane proteins. Several G protein-coupled receptors have been shown to be affected by the cholesterol content of the membrane, with functional impact on their ligand binding and signal transduction characteristics. The effects of cholesterol can be mediated directly by specific molecular interactions with the receptor and/or indirectly by altering the physical properties of the membrane. This review focuses on the importance and differential effects of membrane cholesterol on the activity of cholecystokinin (CCK) receptors. The type 1 CCK receptor is quite sensitive to its cholesterol environment, while the type 2 CCK receptor is not. The possible structural basis for this differential impact is explored and the implications of pathological states, such as metabolic syndrome, in which membrane cholesterol may be increased and CCK1R function may be abnormal are discussed. This is believed to have substantial potential importance for the development of drugs targeting the CCK receptor.

## Introduction

Biological membranes consist of a variety of lipids and proteins that establish diffusional boundaries for the cell and its organelles. An important lipid component of the plasma membrane is cholesterol that may be present in concentrations as high as 20–40 mol%, which is known to have substantial impact on the physical properties of the membrane and on the structure and function of various intrinsic membrane proteins (Mouritsen and Zuckermann, 2004).

The hydrophilic hydroxyl groups of the amphiphilic cholesterol molecules are intercalated into the lipid bilayers, with one cholesterol molecule spanning approximately half of the bilayer (Mouritsen and Zuckermann, 2004). Cholesterol has the unique ability to increase order in such membranes by organizing the arrangement of the surrounding lipids, while maintaining fluidity and lateral diffusion within the membrane. A region of the membrane in which cholesterol is absent typically exhibits disorder and rapid lateral diffusion with randomly packed lipid molecules (Miao et al., 2002; Mouritsen and Zuckermann, 2004). Lowering the temperature can result in a transition from a liquid-disordered phase toward a solid-ordered phase having slower lateral diffusion and greater ordering of the lipid chains. The presence of cholesterol can also induce order in the liquid phase by increasing the density of the packing of fatty acyl chains. This is also identified as a liquid-ordered phase (Ipsen et al., 1987). Cholesterol can also affect the permeability function of the membrane by changing its lateral density fluctuations and structural heterogeneity and thereby regulating its “leakiness.” The effect of cholesterol on lipid order also affects the thickness of the bilayer (Ohvo-Rekila et al., 2002; Mouritsen and Zuckermann, 2004). In the presence of low or absent cholesterol, sodium ions are able to passively permeate the lipid bilayer, while such permeability is inhibited in the presence of high (40%) cholesterol (Ohvo-Rekila et al., 2002). Cholesterol can also bind and inhibit some solutes such as ethanol, which become strongly adsorbed as a result of density fluctuations and the heterogeneous composition of the bilayer (Schroeder et al., 1996).

Cholesterol can also contribute to specialized microdomains within the plasma membrane that are known as planar lipid rafts. These are small, low-density regions in the outer leaflet of the bilayer that are enriched in cholesterol and glycosphingolipids. These structures have been identified based on their insolubility in non-ionic detergents at low temperature and their high buoyancy in density gradients (Brown and Rose, 1992). These planar structures may also give rise to caveolae, representing flask-shaped invaginations that can ultimately form free intracellular organelles (Cohen et al., 2004; Shaw, 2006). It is notable that lipid rafts and caveolae can act as organizational platforms for elements involved in signal transduction (Okamoto et al., 1998; Pike, 2003; Cohen et al., 2004; Ostrom and Insel, 2004).

## Effects of membrane cholesterol on G protein-coupled receptors (GPCRs)

Several GPCRs have been reported to be sensitive to the concentration of cholesterol in the membrane, with different effects observed for different receptors (Table 1). Cholesterol can affect receptor conformation, thereby affecting its ligand binding and signaling characteristics. It can affect lateral mobility within the bilayer that is critical for G protein coupling. It can also affect receptor trafficking and sequestration that contribute to desensitization. However, currently there are no well-established rules for which receptors might be influenced by cholesterol and how these receptors might be affected. Two distinct groups of mechanisms have been proposed: (A) direct binding of cholesterol molecules to GPCR molecules, including the possibility of interacting with specific sites or motifs (Albert et al., 1996; Li and Papadopoulos, 1998; Paila et al., 2009) and/or (B) indirect effects of the cholesterol by altering the physical properties of the membrane in which the GPCRs reside (Lee, 2004; Mouritsen and Zuckermann, 2004).

**Table 1:** **List of GPCRs affected by membrane cholesterol**.

**GPCR**	**Effect of membrane cholesterol**	**References**
β2-adrenergic	Cholesterol improves stability of the receptor and facilitates its crystallization. It also modulates isolation of the receptor from its signaling components in liquid-ordered lipid nanodomains, and thereby affects signaling.	Ben-Arie et al., 1988; Hanson et al., 2008; Pontier et al., 2008
Cannabinoid	CB1R is dependent on cholesterol concentrated in lipid rafts for ligand binding and signaling and the CRAC motif is responsible for its direct interaction with membrane cholesterol.	Bari et al., 2005a,b; Oddi et al., 2011
Chemokine	Cholesterol is essential for CXCR4 and 5 conformation and function.	Nguyen and Taub, 2002a,b, 2003
Cholecystokinin	Cholesterol interacts at specific sites in the transmembrane segments of the CCK1R (CCKAR), and affects its ligand binding and signaling abilities. CCK2R (CCKBR) is not affected.	Gimpl et al., 1997, 2002; Harikumar et al., 2005b; Potter et al., 2012
Dopamine	D1 receptors in renal cells can associate with caveolin-2 in caveolae, where they activate adenylate cyclase.	Yu et al., 2004; Genedani et al., 2010
Galanin	Membrane cholesterol supports the ligand binding process in a positively co-operative manner.	Pang et al., 1999
Metabotropic glutamate	Enrichment of cholesterol in *Drosophila melanogaster* photoreceptor cell membranes induces receptor association with lipid rafts and shift to the high affinity state.	Eroglu et al., 2003
Muscarinic	Cholesterol promotes cooperativity in binding of antagonists to the M2 muscarinic receptors.	Colozo et al., 2007
Neurokinin	Monomeric neurokinin-1 receptors are localized in the lipid rafts and caveolae. Cholesterol content is directly proportional to the signaling.	Monastyrskaya et al., 2005; Meyer et al., 2006
Opioid	Cholesterol present in lipid rafts and caveolae is important for agonist affinity, where it affects G protein coupling.	Lagane et al., 2000; Xu et al., 2006; Huang et al., 2007
Oxytocin	Amount of cholesterol in the membrane is directly proportional to the ligand binding affinity of the receptor.	Gimpl et al., 1997, 2002; Gimpl and Fahrenholz, 2002
Serotonin	Reduction in cholesterol compromises organization, ligand binding, and G protein coupling at the 5HT_1A_ and 5HT_7A_ receptors.	Pucadyil and Chattopadhyay, 2004, 2005; Sjogren et al., 2006
Rhodopsin	Elevated cholesterol inhibits the activation of rhodopsin receptor.	Mitchell et al., 1990; Niu et al., 2002; Bennett and Mitchell, 2008

The effects of membrane cholesterol on rhodopsin, the photoreceptor of the retinal rod cells, have been extensively studied. Rhodopsin exists in various conformations known as metarhodopsins. The equilibrium between these conformational states is sensitive to the amount of membrane cholesterol present, with increased cholesterol shifting the equilibrium toward the inactive states of the receptor (Mitchell et al., 1990; Bennett and Mitchell, 2008). The influence of membrane cholesterol on rhodopsin function has been attributed to both direct and indirect mechanisms. Spatial approximation between tryptophan residues of rhodopsin and cholesterol has been demonstrated using fluorescence resonance energy transfer (FRET), with an estimation of one sterol molecule interacting with one rhodopsin molecule (Albert et al., 1996). These observations have been supported by the crystal structure of metarhodopsin I that includes a cholesterol molecule bound between the tryptophan residues of transmembrane segment four of one protomer and transmembrane segments five, six, and seven of the other protomer (Ruprecht et al., 2004). An indirect effect of cholesterol has also been attributed to its effect on the partial free volume of the membrane. The conversion of metarhodopsin I to metarhodopsin II involves an expansion of the protein in the plane of the lipid bilayer (Attwood and Gutfreund, 1980), thereby occupying the partial free volume of the surrounding bilayer. As membrane cholesterol is increased, the formation of metarhodopsin II is decreased by reducing the partial free volume in the membrane (Niu et al., 2002).

Similarly, cholesterol has been shown to be bound to the β2-adrenergic receptor in its crystal structure (Hanson et al., 2008), and this lipid has been shown to be necessary for ligand binding, G protein interaction, and signal transduction at that receptor (Ben-Arie et al., 1988). Two cholesterol molecules appear to be bound to sites on transmembrane segments one, two, three, and four of a β2-adrenergic receptor molecule (Hanson et al., 2008).

Other examples include the oxytocin receptor, where the amount of cholesterol in the membrane is directly related to the ligand binding affinity of the receptor (Gimpl et al., 1997, 2002; Gimpl and Fahrenholz, 2002) [43% decrease in membrane cholesterol caused a sharp decline in ligand binding (Gimpl et al., 1997)]. This has been shown to be a highly co-operative process where more than six molecules of cholesterol can be bound to one oxytocin receptor molecule (Gimpl et al., 2002). There is clear structural specificity for this interaction, since only cholesterol analogs that are structurally similar to cholesterol are able to reproduce this effect on oxytocin receptor function (Gimpl et al., 1997). Cholesterol is also believed to provide stability against thermal and pH alterations, and to protect some receptors from proteolytic degradation (Gimpl and Fahrenholz, 2002). Similarly, in the case of the galanin-2 receptor, cholesterol affects ligand binding process in a positively co-operative manner (Pang et al., 1999). Only a limited number of cholesterol analogs are able to exhibit similar effects to those of cholesterol, again supporting structural specificity of this interaction (Pang et al., 1999). Other examples of GPCRs affected by membrane cholesterol are listed in Table 1.

Some interactions of membrane cholesterol with GPCRs have been attributed to the presence of consensus motifs within these receptors. Several proteins that are known to interact with cholesterol have a characteristic amino acid sequence, termed the cholesterol recognition/interaction amino acid consensus (CRAC) motif, in their transmembrane segments. This is defined by the pattern—L/V-(X)1–5-Y-(X)1–5-R/K-, in which (X)1–5 represents between one and five residues of any amino acid (Li and Papadopoulos, 1998). This sequence is present in rhodopsin, β2-adrenergic, serotonin_1A_, and cholecystokinin (CCK) receptors (Jafurulla et al., 2011; Potter et al., 2012). Another consensus motif, the strict-cholesterol consensus motif (CCM), was described in transmembrane segments by Hanson et al. (2008), and was later expanded by Adamian et al. (2011). This motif [4.39–4.43 (R,K)] [4.50 (W,Y)] [2.45 (S)] [4.46 (I,V,L)] [2.41 (F,Y)] that incorporates the Ballesteros and Weinstein numbering system based on residue position relative to most conserved residues within a given transmembrane segment (Ballesteros and Weinstein, 1992), was recognized from the analysis of the 2.8 Å structure of the human β2-adrenergic receptor and is present in 21% of the class A GPCRs (Hanson et al., 2008). A less-restrictive variant of this motif is also found in 44% of class A GPCRs, where the aromatic residue is absent at the position 2.41. The presence of this motif, suggests that specific sterol binding may be important to the structure and stability of receptors in this family.

## Cholecystokinin (CCK) peptides and physiology

The GPCRs that are responsive to the gastrointestinal and brain peptide, CCK, are the major focus of this review, with one of the CCK receptors affected by cholesterol and the other unaffected. CCK is a polypeptide hormone synthesized in the I-cells of the small intestine (Rehfeld, 1978) that is released in response to protein and fat in the lumen that plays an important role in nutrient homeostasis. CCK was identified based on its ability to stimulate gallbladder contraction, and it was also eventually recognized as being identical to pancreozymin, a hormone that stimulates pancreatic exocrine secretion (Harper and Raper, 1943). CCK also contributes to post-cibal satiety (Kissileff et al., 1981; Smith and Gibbs, 1985; Beglinger et al., 2001), which is a critically important role that could provide the basis of a treatment of obesity. This hormone is also one of the most abundant neuropeptides present in the brain (Miller et al., 1984), and it has been shown to have effects on enteric smooth muscle and nerves at various locations in the peripheral and central nervous system. It also has been described to have direct natriuretic effects on the kidney, and to decrease renal excretion of calcium and magnesium (Duggan et al., 1988; Ladines et al., 2001). CCK is present as a variety of different length peptides that are produced from a single 115-residue preprohormone precursor, all sharing their carboxyl-terminal sequence. These range from 58, 39, 33, and 8 residues, with each containing a sulfated tyrosine residue seven residues from the carboxyl terminus, as well as amidation of the carboxyl-terminal phenylalanine residue (Eysselein et al., 1990; Rehfeld et al., 2001; Miller and Gao, 2008).

## CCK receptor structure and function

CCK exerts its physiological functions through the activation of two structurally-related class A GPCRs identified as CCK receptor type 1 (CCK1R) and CCK receptor type 2 (CCK2R) (also known as CCKAR and CCKBR, respectively, related to their prominent presence in “alimentary tract” and “brain”) (Dufresne et al., 2006). CCK2R also binds gastrin, another structurally-related polypeptide hormone produced in the gastric antrum (Kopin et al., 1992). In contrast, CCK1R binds gastrin at a very low affinity (by 500-fold). Both these receptors are highly homologous and approximately 50% identical, particularly in the transmembrane regions where they are 70% identical (Miller and Gao, 2008). Although both types of CCK receptors bind and are activated by CCK and gastrin peptides, the molecular basis for peptide binding to these receptors appears to be distinct. The CCK1R requires the carboxyl-terminal CCK heptapeptide-amide that includes the sulfated tyrosine for high affinity binding and biological activity, whereas the CCK2R requires only the carboxyl-terminal tetrapeptide amide that is shared between all CCK and gastrin receptors (Miller and Gao, 2008). The activation of the CCK1R by CCK elicits a broad variety of important physiological functions, such as stimulation of gallbladder contraction and pancreatic exocrine secretion, delay of gastric emptying, relaxation of the sphincter of Oddi, inhibition of gastric acid secretion, and induction of post-cibal satiety (Kerstens et al., 1985; Schmitz et al., 2001). The CCK1R is localized in the human gastric mucosa within D cells (Schmitz et al., 2001) and muscularis propria of human gastric antrum, fundus, and pylorus (Reubi et al., 1997). CCK2R is localized mainly in brain and is also present in the gastric oxyntic mucosa, some enteric smooth muscle, and the kidneys (Noble and Roques, 1999; Von Schrenck et al., 2000). In addition to stimulation of gastric acid secretion, CCK2R also plays a role in anxiety and nociception (Noble and Roques, 1999; Noble, 2007).

The CCK receptors belong to the class A group of GPCRs [see review of CCK receptor structure and pharmacology (Miller and Gao, 2008)]. They share the typical signature sequences of this family, including E/DRY at the intracellular side of transmembrane segment three and NPxxY at the intracellular side of transmembrane segment seven. Both receptors are glycosylated, possess the conserved disulfide bond between extracellular loops one and two (CCK1R also has an extra disulfide bond within the amino-terminal extracellular domain), and include multiple sites for serine and threonine phosphorylation in intracellular loop three and in the carboxyl-terminal tail. The function of phosphorylation is to desensitize the receptor, interfering with its coupling to G proteins (Rao et al., 1997). These receptors also have cysteine residues representing sites of palmitoylation in the intracellular carboxyl-terminal tail, which help to attach an eighth helical segment to the cytosolic face of the bilayer.

A broad range of approaches, including ligand binding and signaling of chimeric and site-specific mutants (Kopin et al., 1995; Miller and Lybrand, 2002), photoaffinity labeling (Ding et al., 2001; Dong et al., 2005, 2007) and fluorescence-based techniques (Harikumar et al., 2004, 2005a, 2006; Harikumar and Miller, 2005) have provided important information regarding the molecular basis of CCK binding to these receptors. Information from these studies has shown that the determinants for CCK binding to the CCK1R are distributed throughout the extracellular loop and amino-terminal tail regions, but not within the predicted transmembrane domain bundle (Miller and Lybrand, 2002; Harikumar et al., 2004). However, a possible difference for CCK binding to these two receptors relates to the position of the carboxyl-terminal end of the peptide. This portion of CCK resides at the surface of the lipid bilayer adjacent to transmembrane segment one for the CCK1R, as directly demonstrated by site-selective photoaffinity labeling (Harikumar et al., 2004), while it may dip into the helical bundle for the CCK2R, although the latter is based on less definitive data coming from site-directed mutagenesis (Harikumar et al., 2006).

Table 2 shows differences in microenvironment-dependent spectral properties of fluorescent CCK ligands docked to CCK1 or CCK2 receptors, supporting the suggested differential binding of the ligands to these two receptors.

**Table 2:** **Summary of studies demonstrating the differential binding of fluorescent CCK probes to CCK1 and CCK2 receptors**.

**CCK1R**	**CCK2R**
CCK-8-based probe had shorter half-life and lower anisotropy (more mobility) in the active than inactive conformation of the receptor. CCK-4-based probe was not tolerated.	CCK-8-based probe had shorter half-life and lower anisotropy (more mobility) in the active than inactive conformation of the receptor. However, when bound, absolute values for lifetime and anisotropy were lower (more exposure to the aqueous milieu) than those in the CCK1R. CCK-4-based probe was tolerated. There was less quenching of this probe (reduced exposure to aqueous milieu) than the CCK-8-based probe.
Fluorophore corresponding to the amino terminus of the CCK (position 24) was more accessible to aqueous milieu in the active than inactive conformations of the receptor. Fluorophore corresponding to the mid-region of the peptide (position 29) was least accessible to the aqueous milieu and unaltered by changes between active and inactive conformations of the receptor. Fluorophore corresponding to the carboxyl terminus of the peptide (position 33) was more accessible to aqueous milieu in the active conformation of the receptor.	Flourophore corresponding to the amino terminus of CCK (position 24) behaved in a similar manner to CCK1R. Fluorophore corresponding to the mid-region of the peptide (position 29) behaved in a similar manner to the CCK1R. The behavior of the fluorophore corresponding to the carboxyl terminus of the peptide (position 33) was less accessible to the aqueous milieu in the active conformation the receptor.

Top, CCK analogs with a fluorescent alexa indicator at the amino terminus of a CCK-8 analog and a CCK-4 analog were used to examine the microenvironment of the fluorophore as docked to CCK1 or CCK2 receptors (Harikumar et al., 2005a).

Bottom, CCK analogs with a fluorescent aladan indicator at the amino terminus, mid-region, or carboxyl-terminus of CCK were used to examine the microenvironment of the fluorophore as docked to CCK1 or CCK2 receptors. Fluorescence quenching, anisotropy and red edge excitation shifts were examined (Harikumar et al., 2006).

Various selective and potent non-natural ligands for the CCK receptor have been developed [see reviews (Herranz, 2003; Kalindjian and McDonald, 2007)]. The most extensively studied in regard to mechanism of binding to CCK receptors is the group of benzodiazepine compounds (Aquino et al., 1996; Darrow et al., 1998; Gao et al., 2008; Cawston et al., 2012). Minor chemical modifications of these compounds have been shown to change receptor subtype selectivity and biological responsiveness (Aquino et al., 1996; Gao et al., 2008; Miller and Gao, 2008). It is now clear, based on receptor mutagenesis, photoaffinity labeling, and pharmacological manipulations, that these ligands bind to an allosteric site within the intramembranous helical bundle that is distinct from the orthosteric CCK peptide-binding site of the CCK1R (Kopin et al., 1994; Hadac et al., 2006; Gao et al., 2008). In fact, using two specific benzodiazepine antagonist ligands, it has been shown that transmembrane segments six and seven [residues 6.51, 6.52, and 7.39 (Ballesteros and Weinstein, 1992)] are most important for binding the CCK1R-selective ligand, whereas residues of transmembrane segments two and seven (2.61 and 7.39) are most important for binding the CCK2R selective ligand (Cawston et al., 2012).

## Effects of membrane cholesterol on CCK receptor function

The ability of CCK to induce gallbladder contraction has been shown to be reduced in individuals with cholesterol gallstones (Fridhandler et al., 1983; Lamorte et al., 1985; Behar et al., 1989; Chen et al., 1997; Amaral et al., 2001; Kano et al., 2002). Initially, it was shown that high cholesterol diet (1–1.2%) induced cholesterol stone formation in the gallbladders of animals like prairie dogs and ground squirrels (Fridhandler et al., 1983). Gallbladder muscle contraction in these models was impaired in response to CCK, but it was also impaired in response to acetylcholine and the non-hormonal stimulant potassium (Lamorte et al., 1985). Studies using muscle strips from human gallbladders with cholesterol gallstones have also demonstrated reduced contractility in response to CCK (Behar et al., 1989). Similar effects were observed when studying *in vivo* gallbladder contraction in response to CCK in patients with gallbladder disease (Amaral et al., 2001). More specifically, using isolated human gallbladder smooth muscle strips and single muscle cells, it has been shown that specimens with cholesterol stones exhibit lower cAMP responses compared with those in gallbladders with pigment stones. Hence, it was suggested that the muscle defect responsible for this impairment was at the level of the plasma membrane (Chen et al., 1997).

Indeed the plasma membrane can incorporate excessive amounts of cholesterol in the presence of cholesterol-rich environment by diffusion (Nichols and Pagano, 1981). Studies measuring the amount of membrane-bound esterified [^3^H]cholesterol in the presence of excessive amounts of unesterified [^3^H]cholesterol, had already confirmed the existence of this phenomenon in cell types such as erythrocytes (Lange and D'Alessandro, 1977) and rat arterial smooth muscle cells (Slotte and Lundberg, 1983). It was suggested from these observations that membrane cholesterol was a key factor in causing the gallbladder muscle impairment. This was confirmed in a study by Yu et al. (1996), in which they measured the cholesterol content of isolated single muscle cells and plasma membranes from gallbladder muscles from prairie dogs, finding an association of elevated cholesterol with reduced muscle contractility. It was reported that after feeding a high cholesterol (1.2%) diet, cholesterol content, and the molar ratio of cholesterol/phospholipid in plasma membranes of gallbladder muscle increased by 90%, with a parallel decrease in muscle contractility by 58% in response to CCK. Similar changes were observed when normal gallbladder muscle cells were incubated with cholesterol-rich liposomes, an effect which was reversed upon incubation with cholesterol-free liposomes (Yu et al., 1996). These observations were also confirmed in human gallbladder muscle from patients with cholesterol gallstones (Chen et al., 1999; Xiao et al., 1999), where the membrane cholesterol content and cholesterol/phospholipid ratio was significantly higher in gallbladders with cholesterol stones than in those with pigment stones. Membrane anisotropy was also higher in gallbladders from patients with pigment gallstones, reflecting lower membrane fluidity in gallbladders from patients with cholesterol gallstones (Chen et al., 1999).

A report studying CCK signaling in isolated single muscle cells from human gallbladders with cholesterol gallstones demonstrated that the production of IP_3_ and diacylglycerol (DAG) was reduced when compared with gallbladders from patients with pigment gallstones (by 80–90% and 78%, respectively) (Yu et al., 1995). However, this effect could be circumvented by stimulating the cells with agents acting directly on G proteins. These results suggested that the defect was proximal to the G protein, at the level of the CCK receptor or its coupling with the G protein (Yu et al., 1995). An illustrative clinical report was published in 1995 that described a patient with morbid obesity and cholesterol gallstone disease in whom the CCK1R was misspliced to yield a non-functional variant missing its third exon (Miller et al., 1995). The frequency of such events is likely extremely low based on another study by the same group (Nardone et al., 1995), in which full length sequencing of the cDNA encoding the CCK1R was shown to be entirely normal in 12 patients with cholesterol gallstones undergoing cholecystectomy (Nardone et al., 1995). Despite normal molecular structure, the function of CCK receptors in patients with cholesterol gallstone disease has been directly shown to be abnormal (Xiao et al., 1999). Of particular interest, the apparent affinity of binding was higher than normal, while the ability of this hormone to stimulate a signaling response was decreased. These abnormalities were reversed after extraction of excess cholesterol from the membrane by incubation with cholesterol-free liposomes (Xiao et al., 1999).

Indeed the impact of membrane cholesterol on this receptor was further explored in manipulatable model systems in 2005 (Harikumar et al., 2005b). This study included extensive analyses of the CCK1R function in lipid-modified environments and showed that normal CCK1R function is dependent on the content of cholesterol in the membrane. In order to monitor the changes in conformation of the CCK1R in the presence of various levels of cholesterol, the study used two different fluorescence-based approaches by measuring anisotropy and lifetimes of a fluorescent full agonist ligand, which shows a decrease in both of these parameters when occupying a receptor in active conformation (Harikumar et al., 2002). Cholesterol depletion from CCK1R-bearing cells using chemical (MβCD chelator) or metabolic (lipoprotein deficient serum supplemented with hydroxymethylglutaryl-CoA reductase inhibitor) methods increased the movement of the label as reflected by decreased anisotropy and reduced lifetime, whereas cholesterol enrichment had the opposite effect. The fluorescence changes in the presence of increased cholesterol were similar to those observed in the presence of a non-hydrolyzable GTP analog which is known to shift the receptor into an inactive, G protein-uncoupled state (Harikumar et al., 2002).

The changes in the conformation of the CCK1R in response to varying amounts of membrane cholesterol are reflected in its ligand binding characteristics and in its biological activity (Harikumar et al., 2005b). Cholesterol depletion was shown to be associated with a reduction in CCK binding affinity, while augmentation of membrane cholesterol content actually increased CCK binding affinity. However, the higher binding in the presence of increased cholesterol was non-productive, resulting in lower biological responsiveness, like the membranes depleted in cholesterol. Notably, the defective intracellular calcium responses to CCK after cholesterol depletion were reversed upon cholesterol repletion. These observations support a defective coupling of the CCK receptor to its G protein in the presence of abnormal membrane cholesterol content (Chen et al., 1997; Xiao et al., 1999).

Other properties such as clathrin-mediated receptor internalization after agonist occupation, and receptor recycling were unaffected by modulation of the membrane cholesterol content, although these regulatory processes have been described to be abnormal after modification of membrane sphingolipid content. This indicates that two different lipid components by themselves or in combination with other lipid components of the plasma membrane probably induce different conformational changes to the CCK1R which can lead to either defective G protein coupling (in case of cholesterol), or disruption in the internalization and recycling pathways (sphingolipid) of the CCK1R. These are reflected by the differences in the observed effects on binding, signaling, and receptor internalization (Harikumar et al., 2005b). However, it is still unclear whether these effects of modification of cholesterol and sphingolipids reflect events occurring in the bulk phase of the plasma membrane or within rafts. Nevertheless, these insights have added a new dimension to our understanding of CCK receptor biology.

## Possible structural basis for the differential sensitivity of CCK receptors to cholesterol

**Figure 1:**
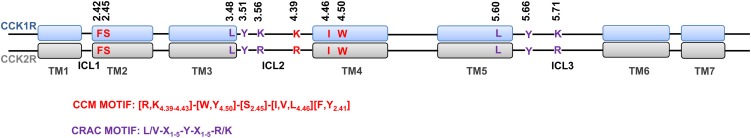
**Schematic representation of the alignment of structural segments of the CCK1R and CCK2R.** The CCM (red) and the CRAC (violet) are highlighted within the transmembrane segments (rectangles) and intracellular loops (black line) of the CCK1R (top, blue) and CCK2R (bottom, gray).

In contrast, the other subtype of CCK receptor, the CCK2R, is not sensitive to alterations in membrane cholesterol (Potter et al., 2012). As noted earlier, these receptors are highly homologous and, in fact, share all of the predicted cholesterol binding motifs (Figure 1). The key structural determinant for cholesterol sensitivity appears to reside in the third exon of the CCK1R which encodes most of the transmembrane segment three and four, including one CRAC motif and one CCM motif (Potter et al., 2012). It should be noted that the CCK1R and CCK2R do not contain the entire four-component strict-CCM, but a less restrictive CCM motif that is shared by 44% of human class A GPCRs (Hanson et al., 2008). This is due to the absence of an aromatic residue at 2.41. The relevant contributors to this effect were further studied with site-directed mutants (Potter et al., 2012). This suggested that three residues, Y140 (Y3.51), which is a part of the CRAC motif (Li and Papadopoulos, 1998) in transmembrane segment three, W166 (W4.50), which is a part of the CCM (Hanson et al., 2008) in transmembrane segment four and Y237 (Y5.66), which is part of the CRAC motif in transmembrane segment five, are important for the cholesterol sensitivity of the CCK1R. It is noteworthy that mutation of each of the three residues has negative effects on either CCK binding or CCK-induced signaling. Mutation of Y140 to alanine leads to reduced signaling but increased CCK binding affinity and is insensitive to reduction in membrane cholesterol levels. On the other hand, mutation of W166 to alanine leads to decreased CCK binding and signaling ability of the CCK1R, with a further reduction in signaling upon depleting membrane cholesterol. The Y237A mutant displays no change in signaling, but reduced CCK binding affinity, whereas cholesterol reduction results in the reduction of both parameters. Corresponding single residue mutations (Y153A, W179A, Y246A) within the CRAC and the CCM motifs of the CCK2R did not modify receptor function.

It is remarkable that mutation of only Y140 residue within the CRAC motif within transmembrane segment three resulted in loss of cholesterol sensitivity of the CCK1R (Potter et al., 2012). This implies a defective conformation of the receptor, particularly because the mutation alters the conserved (D/E) 3.49-R3.50-Y3.51 motif, and thus its ability to mediate the differential effects of cholesterol in the two structurally related receptors is compromised. However, studies with other class A GPCRs have shown that the mutation of Y3.51 of the D/ERY motif does not affect the ligand binding affinity or receptor trafficking, and has no or marginal effects on receptor signaling (Ohyama et al., 2002; Gaborik et al., 2003; Proulx et al., 2008). So, it appears that probably this residue is specifically responsible for the differential cholesterol sensitivity observed between the CCK receptors. This implies that the transmembrane segment three CRAC cholesterol binding motif could be responsible for the cholesterol sensitivity of the CCK1R, because the transmembrane segment four CCM (W166) and transmembrane segment five CRAC (Y237) mutants are still sensitive to cholesterol. However, the transmembrane segment three CRAC cholesterol-binding motif is essentially the same in both the CCK receptors, differing only in the presence of lysine in CCK1R and an arginine in the CCK2R. Both the residues which are a part of the consensus motif have similar properties. While the sequence surrounding the motif is largely similar, they do exhibit minor differences between the two receptors that could contribute to the observed difference in the cholesterol sensitivity.

These observations show that the presence of a CRAC or/and CCM motif do not necessarily mean that the receptor is sensitive to cholesterol. At least in the case of the CCK receptors, it appears that the subtype specific conformational changes are conferred by the differences in direct interaction of the membrane cholesterol with the two receptors. It has been shown in the case of the human cannabinoid receptors (CBR), that the type 1 CBR (CB1R) is affected by manipulations in the membrane cholesterol content, in a way similar to the CCK1R exhibiting defective ligand binding and signaling, as well as its localization to membrane rafts (Bari et al., 2005a,b). On the other hand, similar to CCK2R, the type 2 CBR (CB2R) does not share this sensitivity to membrane cholesterol (Bari et al., 2005a,b). In fact it was found that CB1R possesses a CRAC motif in transmembrane segment seven that differs by one residue from that in the CB2R, and the corresponding mutation rendered CB1R insensitive to membrane cholesterol similar to CB2R (Oddi et al., 2011). This explains that cholesterol binding motifs outside of the CCM can have a significant influence on receptor signaling and trafficking.

In addition to this, other possibilities for the differential sensitivity of CCK receptors may be related to the indirect impact of the cholesterol on the processes like mechanism of ligand binding or receptor trafficking. As mentioned earlier, the CCK1R and the CCK2R bind the same CCK peptide ligand differently (Silvente-Poirot and Wank, 1996; Dong et al., 2005; Harikumar et al., 2005a, 2006). The peptide ligand binds to extracellular loops and the amino-terminal tail of the CCK1R, whereas in the CCK2R, the carboxyl-terminal end of the peptide ligand may dip into the helical bundle (Harikumar et al., 2005a, 2006). It can be postulated that in the case of CCK2R the peptide binding within the helical bundle could potentially stabilize it and consequently the intracellular regions as well, and prevent the negative impact of changing membrane cholesterol. On the other hand, the impact of membrane cholesterol cannot be overcome in the case of the CCK1R because the conformational changes affecting the extracellular regions binding the ligand are very different to that conferred to the part of the receptor within the lipid bilayer. So, these differential changes on the domains of the receptors which are physically further away, may dictate the observed effects of cholesterol sensitivity in the CCK1R.

Other processes occurring post ligand binding can also be affected by changes in membrane cholesterol, which can contribute to the observed effects. In the case of CCK1R, the receptor has been described to occupy a unique plasma membrane compartment after CCK stimulation of rat pancreatic acinar cells (Roettger et al., 1995). This was called as a site of “insulation” that is comparatively devoid of G proteins, in which the lateral mobility of the CCK receptor is markedly reduced as another highly specialized cellular mechanism of desensitization (Roettger et al., 1999). Accordingly, it can be postulated that the membrane cholesterol can cause similar changes to the CCK1R, however, there is no information yet about the existence of a similar mechanism for CCK2R. Also, whether the CCK receptors are located in the lipid rafts, and if the cholesterol modulation effects are exclusive to these regions remains to be investigated.

## Conclusion and therapeutic perspectives

It is clear that lipid-protein interactions, especially cholesterol-receptor interactions, involved in the function of GPCRs can be extremely important. More specifically, this review has focused on the role of cholesterol in the function of the CCK1R, an important receptor likely to be involved in pathologic states such as obesity and metabolic syndrome. A better understanding of the mechanisms of appetite regulation that contribute to the development of obesity could provide insights into the prevention and more effective treatment of this disorder. The gastrointestinal hormones like CCK play important roles in servomechanisms involved in regulating nutritional homeostasis. The observations showing that elevated membrane cholesterol reduce the effectiveness of stimulus-activity coupling activated by CCK action at the CCK1R provide vital information regarding the pathological phenomenon associated with CCK receptor dysfunction. It is essential and would be very interesting to study the membrane environment of the CCK1R receptor in obese patients, in order to verify these observations in humans. Additionally, it would be very exciting to study whether the mutations affecting the cholesterol sensitivity might also be present in these patients. Overall, the observations so far may suggest that elevated levels of membrane cholesterol described in cholesterol gallstone disease and metabolic syndrome could reduce hormone-stimulated signaling and, thereby, reduce the feedback inhibition of food consumption that is an essential servomechanism. If this is proven true in patients, the CCK1R can become the target for future development of positive allosteric modulators that could “recalibrate,” and consequently “normalize” this important regulatory mechanism.

### Conflict of interest statement

The authors declare that the research was conducted in the absence of any commercial or financial relationships that could be construed as a potential conflict of interest.

## Abbreviations

CCK: cholecystokinin
cAMP: cyclic adenosine monophosphate
CCM: cholesterol consensus motif
CRAC: cholesterol recognition/interaction amino acid consensus
DAG: diacylglycerol
FRET: fluorescence resonance energy transfer
GPCRs: guanine nucleotide-binding protein-coupled receptors
MβCD: methyl-beta-cyclodextrin
IP_3_: inositol triphosphate.
